# Validation of the Revised Knox Preschool Play Scale for the Brazilian Population

**DOI:** 10.1155/2019/6397425

**Published:** 2019-05-02

**Authors:** Amanda Mota Pacciulio Sposito, Jair L. F. Santos, Luzia Iara Pfeifer

**Affiliations:** ^1^Ribeirão Preto College of Nursing, University of São Paulo, Ribeirão Preto, Brazil; ^2^Ribeirão Preto Medical School, University of São Paulo, Ribeirão Preto, Brazil

## Abstract

**Background:**

Play is essential to child development, and its evaluation is considered valid to indicate the stage of development of the child and indicate possible lagging. The Revised Knox Preschool Play Scale (RKPPS) provides an evolving description of the typical play behavior of preschool children, in six-month periods from zero to three years of age and then in annual periods up to six years of age. The RKPPS has already undergone the process of cultural adaptation for use in the Brazilian population, and it is necessary to analyze its applicability.

**Aims:**

To verify the reliability and internal consistency of the RKPPS for Brazilian children.

**Method:**

135 children participated in the study, divided into different age groups with 15 in each group, and were filmed during free play in school or home contexts. Two independent raters evaluated the footage from two different times. Based on these evaluations, a statistical analysis was carried out in order to ascertain the reliability and the internal consistency of the Brazilian version of the RKPPS.

**Results:**

Intra- and interrater reliability showed a predominance of near-perfect to moderate agreement; however, some dimensions of certain age groups presented reasonable to poor agreement. The internal consistency was found to be satisfactory for most of the items evaluated; however, there were items with poor results in some dimensions of certain age groups.

**Conclusions:**

There is a need for further analysis of these items by a committee of experts to ensure the reproducibility of the instrument.

## 1. Introduction

Play is indispensable at the preschool age as it stimulates social, cognitive, emotional, physical, sensory, and language developments [1]. More specifically, it enables the understanding and assimilation of culture, as well as learning and problem solving, allowing for the integration of environmental information, the construction of mental representations, and greater flexibility of thought [2–4].

Playful activity connects and creates bonds between participants, even if this is only temporary [5]. Through play, the child can communicate fear and anxiety, acquire a sense of control of the situation [6], and experience different feelings, such as joy, success, and frustration; these are diverse experiences that will aid in the structuring of the personality.

Play allows the child to test and develop their skills, as well as to stimulate creativity, initiative, and self-confidence [7]. It prepares the individual for their future work activities, in that it favors the development of attention and concentration, stimulates self-esteem, and helps to develop relationships of trust. It also helps the child explore their relationship with the world, sharing spaces and experiences with other people [8].

As play is essential and intrinsically related to child development, it is possible to evaluate the stage of development of a child and any potential delay by using a play assessment. Thus, standardized instruments that evaluate play can be used to determine the child's eligibility for therapeutic service, monitor progress achieved throughout the treatment process, and assist in decisions about the most appropriate and effective intervention for the case, as well as provide a common language among professionals which helps facilitate their communication with the family [9].

In 1968, the American occupational therapist Susan Knox developed a play scale. At the time, the Scale was used to evaluate children with mental retardation; however, no normative data were collected nor was the reliability and validity verified [3].

Bledsoe and Shepherd [10] reviewed the instrument and renamed it the Preschool Play Scale, proposing minor changes to its content to make all items mutually exclusive, maintain consistency between categories and age groups, and update the Scale, incorporating data from recent studies from the period. After these modifications, they verified its reliability and validity, which was later confirmed by Harrison and Kielhofner [11].

Other studies used the Preschool Play Scale to evaluate different infant populations [12–17], and from the experiences of these authors, Susan Knox was able to identify positive and negative points in her instrument and then proposed the Revised Knox Preschool Play Scale (RKPPS).

This is a rating scale, based on observation, which provides an evolving description of the typical play behavior of preschool children, in periods of six months from zero to three years of age and then in annual periods up to six years of age. In each age group, the actions and behaviors that children normally present when playing are described and are divided into four dimensions: space management, material management, pretense/symbolic, and participation [3].

Considering the potential clinical and academic usefulness of the RKPPS, the transcultural adaptation of this instrument for use in the Brazilian population was performed [18], as well as a pretest of this version in Portuguese [19], and the statistical analysis indicated reliability, consistency, and repeatability of the Brazilian version of the RKPPS [18, 19]. The purpose of the present study was to validate the construction and content of the Revised Knox Preschool Play Scale for application in the Brazilian population.

## 2. Materials and Methods

The current study is an applied, nonexperimental, cross-sectional design, using quantitative analysis research which was carried out in nurseries/daycare facilities and public and private preschools of two municipalities in the state of São Paulo, whose children were recruited, randomly, by the teachers of the classroom to be included in the research. As this research has a convenience sample, infants who met the inclusion criteria (presented below), but were not linked to any educational institution, were assessed at the children's own home.

### 2.1. Participants

A total of 135 children participated in this study, with 15 children in each of the following age groups (according to the division established by the RKPPS): 0 to 6 months, 6 to 12 months, 12 to 18 months, 18 to 24 months, 24 to 30 months, 30 to 36 months, 36 to 48 months, 48 to 60 months, and 60 to 72 months. The sample size was calculated to allow, in each age group, to be able to detect differences of up to 25%, in a one-tailed test under maximum variability, with a significance level of alpha of 10% and test power of 77% [20].

The following inclusion criteria established were children without motor, cognitive, and sensory impairment; aged between 0 and 72 months; and who had the authorization of parents or legal guardians, which was obtained by signing the consent form, to participate in the research.

### 2.2. Data Collection

For the data collection, the children were filmed playing freely, according to the original guidelines of the application of the RKPPS [3]. Babies from 0 to 6 months and 6 to 12 months were filmed individually, for 30 minutes in an enclosed, yet spacious, location. During a few minutes of filming, the child could interact with the caregiver, enabling the analysis of the RKPPS “participation” dimension. The inclusion of the caregiver during the data collection was always explained and agreed, what they could or not do, prior to the beginning of the recording. However, this caretaker was not considered a study participant (but rather a research assistant) and was not considered in the analysis.

Children aged 12 to 18 months, 18 to 24 months, 24 to 30 months, 30 to 36 months, 36 to 48 months, 48 to 60 months, and 60 to 72 months were observed in pairs or trios. The filming of these age groups was performed in two different environments, 30 minutes in a closed room and 30 minutes on the outside, allowing for engagement in different types of games. Toys designed for the motor, sensory, and pretend plays were made available, according to the age range of the evaluated child, to stimulate free play.

### 2.3. Data Analysis

In the pretest of the Brazilian version of the Scale [19], the scoring proposed by Pfeifer [21] was successfully used, as well as in the preliminary manner in other studies [22–25], showing high correlation coefficient and good significance levels of the reliability and repeatability of the instrument.

Thus, as a way to quantify the behavior and performance of the children participating in this study, in each item observed in the filming, this scoring proposal by Pfeifer [21] was used, which establishes

- if the child confidently presents the expected behavior or performs the task satisfactorily: 2 points
- if the child does not confidently present the expected behavior or hesitantly performs the given task: 1 point
- if the expected behavior or the task determined could not be observed due to no interest of the child to play or the absence of specific toy or play equipment: 0 point
- if the child did not present the expected behavior or did not perform the given task, even when having the opportunity to do so: -1 point

The intra- and interrater reliability was assessed using the Cohen kappa coefficient [26], and the internal consistency of items and dimensions of the instrument was analyzed using Cronbach's alpha [27]. Two raters (undergraduate students of occupational therapy, previously trained to apply and score the RKPPS) watched the filmed footage separately from two different times (at least 3 months apart). Landis and Koch [26] suggest that the interpretation for the kappa values found in the analyses is as follows: kappa value < 0 = without agreement, 0 − 0.19 = poor, 0.20 − 0.39 = reasonable, 0.40–0.59 = moderate, 0.60 − 0.79 = substantial, and 0.80 − 1.00 = almost perfect. Tavakol and Dennick [28] report that satisfactory values of Cronbach's alpha range from 0.70 to 0.95.

## 3. Results

**Figure 1:**
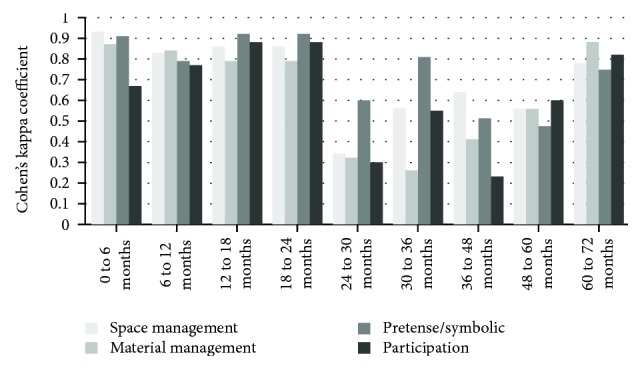
Intrarater agreement for rater 1.

**Figure 2:**
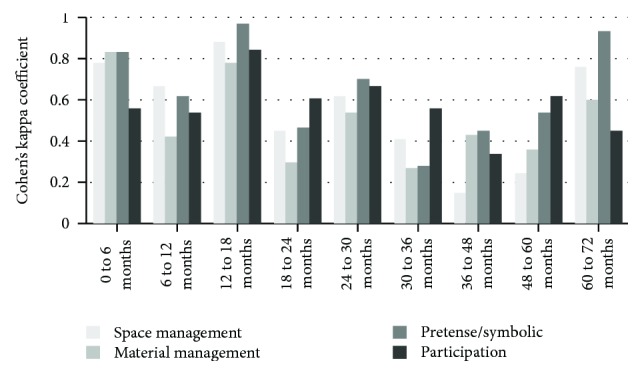
Intrarater agreement for the rater 2.

**Figure 3:**
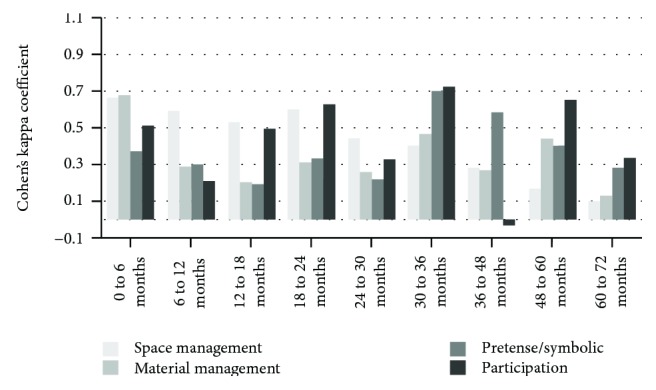
Interrater agreement.

Figures 1–3 present the results of the reliability analyses using Cohen's kappa coefficient of the intra- and interraters in each of the 9 age groups that compose the RKPPS.

It could be verified that the degree of agreement between the evaluations of rater 1 was good in most dimensions and age groups, with a predominance of near perfect agreement and substantial agreement for 10 topics each, followed by moderate agreement on 9 topics; however, 7 topics presented reasonable agreement.

Analyzing the degree of agreement for the evaluations of rater 2, there is a predominance of substantial and moderate agreements for 10 topics each, 6 with almost perfect agreement; however, there were 7 topics with reasonable agreement and 1 with poor agreement.

The interrater agreement had a predominance of reasonable agreement, with 13 topics, while another 4 topics presented poor agreement, which suggests a need to review the items involved in the dimensions evaluated.

**Figure 4:**
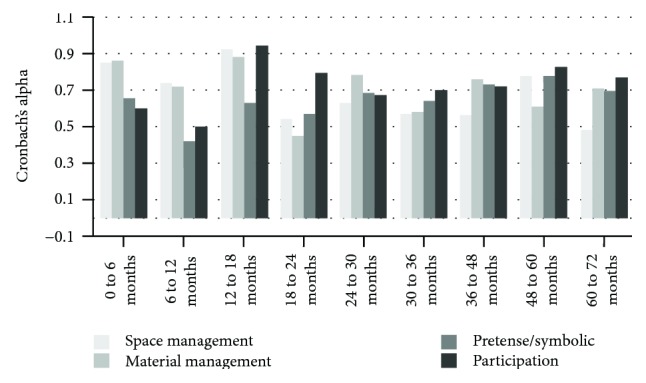
Internal consistency for rater 1.

**Figure 5:**
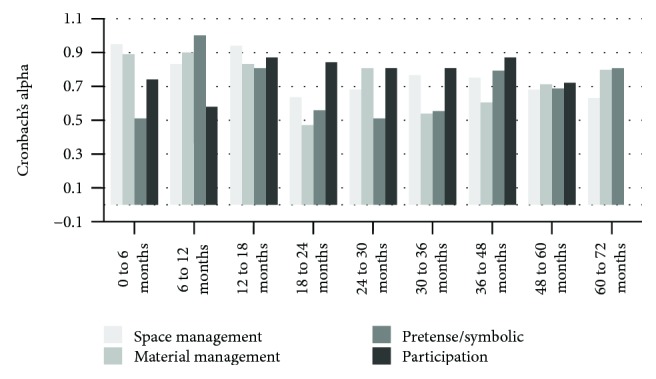
Internal consistency for rater 2.

Figures 4 and 5 present the results of the analyses using Cronbach's alpha to verify the internal consistency of each one of the domains of the age ranges that compose the Scale.

More than 50% of the topics analyzed have good internal consistency (19 out of 36 dimensions evaluated) with Cronbach's alpha value range from 0.70 to 0.95.

Here as well, most of the items presented good internal consistency (23 out of 36 item dimensions evaluated) with Cronbach's alpha value range from 0.70 to 0.95.

## 4. Discussion

The process of cross-cultural adaptation promotes communication between different researchers and the comparison of data obtained at an international level, as well as being considered faster and economical than the development of new instruments [29, 30]. After the stage of translation and cultural adaptation, the validation of an instrument for use in a given population should be performed [31].

The content validation verifies if each element of the instrument is relevant and representative within the purpose of this assessment, and for this, a multidisciplinary committee, composed of bilingual persons and specialists in the area of the evaluation instrument, analyzed the adapted version [31]. Following these procedures, the Brazilian version of the RKPPS has already undergone a preliminary process of validation of content [18].

Despite this process, the reliability analysis of the intrarater and interrater evaluations using the Cohen kappa coefficient revealed that some domains in each of the age groups of the RKPPS presented reasonable or poor agreement. Both raters evaluated each child by video footage; therefore, they did not have contextual or time/day differences between their assessments. These results indicate that adjustments are still needed in some items of the Brazilian version of RKPPS so that this instrument can be utilized confidently by Brazilian professionals to provide a reliable assessment of children's play performance over time. Thus, the Brazilian version of the RKPPS should have a new content analysis, involving a committee of experts, to analyze in detail each item within the domains in which there was disagreement between the raters. The experts can then identify which items require more appropriate definitions of the behavior to be evaluated and which of them need more detailed explanation exemplifying how to proceed with the assessment.

The internal consistency presented some domains with Cronbach's alpha values lower than 0.70 and others higher than 0.95, which is not considered satisfactory [28]. This is a limitation of the present study as these areas with unsatisfactory values must also undergo a new detailed evaluation with a committee of experts, identifying the items that need changes.

Finally, it is suggested that, once this content validation process is completed, an orientation manual for applying the Brazilian version of RKPPS should be prepared, to clarify and define the application and evaluation for each item, providing therapists with more detail of the specificities that need to be observed, assuring the reliability of this assessment.

## 5. Conclusions

There have been many efforts in the validation process of RKPPS for use in the Brazilian population. However, despite these, the statistical analysis of the reliability and internal consistency of its domains has indicated the need for further and deeper analysis; in addition, there is also the need to identify items that generate disagreements in the evaluations and interfere with the reproducibility of the instrument.

## Data Availability

The complete data will be available, at the doctorate thesis, on July 2019 on the website http://www.teses.usp.br/.
